# 

**Published:** 1896-05

**Authors:** 


### AMERICAN JOURNAL

OF

### DENTAL SCIENCE,

17

EDITED BY

F. J. S. GORGAS, M. D., D. D. S.,

RICHARD GRADY, M. D., D. D. S.

VOLUME XXX—THIRD SERIES.

(1896-7.)

BALTIMORE:
SNOWDEN & COWMAN MFG. CO.

SURGEON GENL'S OFFICE
158159
LIBRARY.

LONDON:
TRUBNER & CO., PATERNOSTER ROW.
1897.

### Index to Vol. XXX---Third Series.

A Mechanical Horror ........ 43
Advantages of Pyrozone ........ 46
Administering Nitrous Oxid ........ 47
Anæsthesia, Local and General ........ 49
American Dental Association ........ 87
Anæsthesia, Local and General ........ 97
Air Chambers ........ 118
A Case of Removal of the Major Portion of the Lower Jaw for Sarcoma ........ 126
Acute Abscess of Teeth ........ 129
Abstracts from a Discussion Following the Reading of a Paper upon "Soft Foil and Conservative Methods" ........ 171
Alloy and Cement for Filling ........ 183
Amalgam Filling ........ 185
Amalgamating Gold Sections into Teeth ........ 186
A Cheap Hydrogen-gas Flash Lamp ........ 192
A Plea for the Higher Scientific Education of the Individual Members of the Profession ........ 193
A Local Obtundent ........ 236
A Remedy for Thirst ........ 238
A Half Century Mark in Dental Education ........ 240
A Substitute for Gold ........ 240
A Talk on Materia Medica ........ 241
A New Method of Doing Bloody Operations ........ 288
A Plan for a National Dental Association Divided into Four Co-ordinate District Branches ........ 354
A Word of Caution ........ 378
A Valuable Hint ........ 378
A History of Maryland's New Dental Law ........ 463
A Case of Pyorrhea Alveolaris ........ 476
A New Test for Albumen ........ 478
Attachment of Logan Crown ........ 479
A New Hemostatic ........ 480
Another Source of Infection ........ 480
A Plea for Conservative Oral Surgery with Practical Illustrations ........ 481
A Criticism of the Editorial "Dental Faculties in Medical Schools" ........ 517
A Good Prescription ........ 528
An Indian Has a Tooth Filled ........ 528
Balsam Varnish ........ 46
Bad Breath ........ 144
"Borism," or the Toxic Effects of Borax ........ 204

Bacteriologists........................................ 239
C. E. Bentley, in Items of Interest .................... 45
Chronic Abscess.... . .................................. 61
Cataphoresis for Obtunding Sensitive Dentin............. 120
Cohesion and Adhesion................................... 137
Carbolic Acid........................................... 141
Chronic Gastritis of Long Standing, with Periodic Attacks of Migraine...... ........................................ 143
Calcareous Deposits During Pregnancy ................... 144
Chlorate of Potash as an Antiseptic and Germicide for the Mouth .................................................. 156
Calomel Treatment in Typhoid Fever...................... 191
Cataphoresis............................................ 192
Cataphoresis...... ..................................... 206
C. D. Hertz, in Items of Interest....................... 236
Cuspid Angles........................................... 238
Cancrum Oris............................................ 252
Capping Pulps........................................... 286
Congenital Teeth........................................ 380
Chicago Dental Society.................................. 423
Cataphoresis Simply Explained........................... 429
Cataphoresis............................................ 494
Committee for Tri-union Meeting of the Washington City Dental Society, Maryland State Dental Association and the Virginia State Dental Association, 1897.............. 520
Chloroform.............................................. 527
"Cataphoresis". ........................................ 527
Cataphoresis in Dental Practice......................... 539
Cataphoretic Extirpation of Living Pulps................ 452
Creosote ............................................... 576
Dentistry in Japan. .. ................................. 5
Dentistry a Distinct and Independent Profession.... ....... 22
Dentistry in Brazil..................................... 41
Dr. George S. Fouke..................................... 92
Dr. Felix E. Brown...................................... 93
Duration of Pulpless Teeth.............................. 124
Dental Hemorrhage ...................................... 141
Dr. Genese on an Efficient Obtundent ................... 184
Dark Joints in Vulcanite Plates—How to Prevent Same...... 225
Dr. C. W. Spalding...................................... 284
Dr. H. N. Wadsworth..................................... 285
Dental Pain...... ...................................... 333
Dental and Medical Infection............................ 337
Death Three Days After Chloroform Administration........ 384
Dental Inspectors for Schools........................... 384
Dr. Jack's Views on Implantation ....................... 430
Dentistry in the Orient.................................. 433

Dental Sinners........ 477
Diagnosis of Pulpitis........ 523
Diphtheria and Scarlet Fever........ 555
Dentists in the U. S. Army........ 565
Electricity in Dental Practice........ 12
Empyema of the Antrum of Highmore ........ 258
Eucain........ 455
Edison Current in Cataphoresis........ 513
Empyema of the Antrum of Highmore........ 541
Filling Pulpless Teeth with Fistulous Opening........ 47
Filling a Cavity Temporarily........ 48
For Separating Teeth........ 237
For Sensitive Dentine........ 239
Flexible Rubber Edges........ 331
Filling of Canals and Pulp Cavities........ 385
Formalin in Dental Practice........ 428
Foreign Dentists in Hungary........ 432
Gold Fillings........ 215
Green stain........ 329
Hydrozone in Purulent Otitis Media........ 94
How to Disguse the Taste of Cod Liver Oil........ 237
Hypnotism........ 430
Hygiene of the Mouth........ 473
Inter-State Medical Certificates........ 91
Illinois State Dental Society........ 86
Insomnia........ 142
Implantation........ 178
In Anesthesia........ 192
Infiltrated Anesthesia in Dentistry........ 328
Interesting Experience........ 379
Influence of Condiments on Digestion........ 380
Investing Material........ 383
Itching in Eczema........ 479
Ice to Relieve Dyspepsia........ 480
Illinois State Dental Society........ 568
James Taylor, M. D., D. D. S........ 135
Local Anesthesia........ 10
Local Anesthesia and Obtundents........ 78
Local Anesthetic........ 186
Lining for Rubber Plates........ 332
Local Anesthetic........ 335
Long Operations........ 336
Longevity of Human Life Increasing........ 478
Liquid Silex........ 479
Making Crowns........ 15
Maryland Dental Law........ 88
Medical Hints........ 234

Method of Verifying the Qualify of Alcohol .......... 237
Masticating .......... 287
Metal Dies Direct from Impression .......... 476
"Murder (and Other Crimes) Will Out" .......... 528
Moses Atwood Hopkins, D. D. S .......... 572
National Association of Dental Faculties .......... 85
Notes Upon Dentine and Enamel .......... 106
No Toothache in that Factory .......... 142
New Jersey State Dental Society .......... 182
New Treatment for Pyorrhea .......... 184
Nerve Tension .......... 189
National Association of Dental Faculties .......... 274
New Jersey State Dental Society .......... 294
Novel operation on the Eye .......... 349
New Local Anæsthetic .......... 379
Nitrate of Silver .......... 524
Nuclein in Dental Practice .......... 526
Nodule on Apex of Root .......... 575
On "Light" .......... 317
Odontographic Society of Chicage .......... 424
Obtunding Sensitive Dentine .......... 428
Oral Surgery .......... 458
Photographing the Unseen .......... 1
Porcelain Inlays .......... 7
Pink Base Plate .......... 48
Preparation of Pulp-Cavity and Canals .......... 67
Pulp Protection in Filling Teeth .......... 72
Peroxide of Hydrogen .......... 96
Practical Laboratory Points .......... 159
Percentage Solutions .......... 186
Prevention of Infectious Diseases .......... 233
Pains After Extraction of the Teeth .......... 255
Pulpless Teeth, Roots and Their Treatment .......... 271
Preparation of Cavities .......... 330
Preparing a Tooth for Porcelain Crown .......... 332
Peculiar Affection of the Mucous Membrane of the Lips and Mouth .......... 383
Points of Interest in the Care of Children's Teeth .......... 410
Preparing a Tooth for a Porcelain Crown .......... 429
Pathology and Therapeutics of Dead Teeth .......... 449
Pyorrhea Alveolaris From a New Standpoint .......... 502
Pulpitis vs. Peridontitis .......... 526
Pulpitis .......... 527
Pyorrhea Not Infectious .......... 553
Roentgen's Discovery .......... 33
Relief of Headache Following Dental Operations .......... 45
Repairing Broken Down Teeth .......... 139

Root Canal Cleansers.................................... 187
Root Sterilization.................................... 240
Remedial Hypnotism.................................... 265
Retaining Artificial Dentures in the Mouth.................................... 269
Roentgen Rays—Their Application to Medicine and Surgery.................................... 307
Resuscitation From Electric Shock.................................... 334
Report of the Committee on National Dental Museum and Library.................................... 419
Requirements to Practice Dentistry in Foreign Countries.... 424
Specific Treatment of Necrosis of the Alveoli and Maxillæ with Aromatic Sulphuric Acid.................................... 17
Some Practical Points in Porcelain Work.................................... 36
Saving Teeth.................................... 43
Spot Specialism.................................... 47
Specific Remedies.................................... 48
Saw His Own Heart.................................... 82
Solid Cusps.................................... 161
Soldering.................................... 164
Skiagraphy in Dentistry.................................... 182
Some Suggestions on Bridge-work.................................... 199
Swaging.................................... 287
Suggestions of Crown-work.................................... 357
Some Facts About the Automatic Mallet.................................... 401
Surgery of the Maxillary Sinus.................................... 404
Simplified Treatment of Teeth with Exposed Pulps.................................... 406
Smoking and Intellectual Labor.................................... 432
Shall We Ask Congress for a Special Law Regulating Medical and Dental Patents?.................................... 545
The Relations of Materia Medica and Therapeutics in Stomatology.................................... 27
To Sterilize Steel Instruments Without Reducing their Temper.................................... 44
The National Association of Dental Examiners.................................... 87
The Chicago Dental Society.................................... 86
The American Dental Society of Europe.................................... 86
Treatment of Abscessed Teeth.................................... 132
Test for Insane Persons.................................... 134
Tooth in Her Lung.................................... 138
The Advertising Dentist.................................... 140
Tobacco from an Esthetic and Hygienic Point of View.................................... 145
Treatment of Root Canals for Immediate Filling.................................... 188
To Bore Glass.................................... 188
The House We Live In.................................... 189
The Truth About Bacteriology.................................... 190
Treating Dead Teeth.................................... 192
The Physical Characteristics of the Teeth.................................... 213

The Successful Dentist.................................... 217
The Future of Dentistry.................................... 220
Treatment of Decay in Proximate Surfaces of Bicuspids and Molars.................................... 226
The Philadelphia Polyclinic.................................... 232
The Life and Growth of the Teeth.................................... 235
Teeth of Old Age.................................... 248
The Spitting Habit.................................... 250
The Dental Filling.................................... 289
The Soldering Iron.................................... 331
The Greek Surgeons.................................... 333
Tin and Gold Filling.................................... 334
To Clean and Remove Stains from Teeth.................................... 334
The Saving of the Pulp.................................... 335
Treatment of Abscess.................................... 335
The Human Voice.................................... 336
The Army Medical Museum and Library.................................... 340
The Life and Growth of the Teeth.................................... 352
The Dental Society of the State of New York.................................... 359
The Physical Characteristics of the Teeth.................................... 375
Treatment of Antrum Disease.................................... 377
Toothache Drops.................................... 381
Treatment of Pulpless Teeth.................................... 384
The Use of Roentgen Rays.................................... 431
Temporary Stopping.................................... 432
Taking Impressions and Fitting Artificial Dentures.................................... 446
The Investigation of Uric Acid in Salivary Tartar in the Course of Pyorrhea Alveolaris (Artaro-Dental Infectious Gingivitis.................................... 452
The Bicycle and the Perineum.................................... 471
The Orbicularis Oris and the Muscles of Expression.................................... 529
The Latest Discovery.................................... 543
The Inheritance of Mutilations and other Inheritances.................................... 559
The Mouth Mirror.................................... 562
The Baltimore College of Dental Surgery.................................... 570
University of Buffalo, Dental Department.................................... 134
University of Maryland, Dental Department.................................... 568
Verses by Dr. Evans.................................... 41
Why are Our Teeth Degenerating.................................... 65
Why are We Right-Handed.................................... 75
What We Need.................................... 140
With a Poisoned Lancet.................................... 239
Why Amalgams Fail.................................... 381
White Caries.................................... 382
When to Fill.................................... 416
Whooping Cough Bacillus.................................... 528

### To Readers and Correspondents.

Communications intended for publication, and exchange journals and papers, should be addressed to the Editor of The American Journal of Dental Science, No. 845 N. Eutaw St. Baltimore, Md. All letters relating to the business of the Journal, or containing cash remittances, to be directed to Snowden & Cowman Mfg. Co., 9 W. Fayette Street, Baltimore, Md. Articles for publication should be received by the Editor at least two weeks previous to the first of the month on which the number in which it is designed for them to appear is issued.

Verses by Dr. Evans. - - - - - - - - 41

### MONTHLY SUMMARY.

Dentistry in Brazil. - - - - - - - - 41

A Mechanical Horror. - - - - - - - - 43

Saving Teeth. - - - - - - - - - 43

To Sterilize Steel Instruments Without Reducing their Temper. - 44

C. E. Bentley, in Items of Interest. - - - - - 45

Relief of Headache Following Dental Operations. - - - 45

Advantages of Pyrozone. - - - - - - - 46

Balsam Varnish. - - - - - - - - - 46

Filling Pulpless Teeth With Fistulous Opening. - - - 47

Spot Specialism. - - - - - - - - - 47

Administering Nitrous Oxid. - - - - - - - 47

Pink Base Plate. - - - - - - - - - 48

Specific Remedies. - - - - - - - - - 48

Filling a Cavity Temporarily. - - - - - - - 48

☞ The American Journal of Dental Science will contain fifty pages of reading matter in each number, making a volume of about Six Hundred pages,

EXCLUSIVE OF ADVERTISEMENTS.

